# Comparative Analysis of Dentition and Periodontal Status in Patients With Unilateral Smokeless Tobacco Pouch Keratosis

**DOI:** 10.7759/cureus.48923

**Published:** 2023-11-16

**Authors:** Anand V Nimbal, Shardha P Kharkar, Aruna P Vishwakarma, Asmita A Patil, Snehal S Patil, Rutuja A Patil

**Affiliations:** 1 Department of Dentistry, Shri B. M. Patil Medical College Hospital and Research Centre, Vijayapura, IND; 2 Department of Dentistry, Ex-Servicemen Contributory Health Scheme (ECHS) Polyclinic, Dhule, IND; 3 Department of Pediatric Dentistry, Jawahar Medical Foundation's Annasaheb Chudaman Patil Memorial (JMF's ACPM) Dental College, Dhule, IND; 4 Department of Public Health Dentistry, Jawahar Medical Foundation's Annasaheb Chudaman Patil Memorial (JMF's ACPM) Dental College, Dhule, IND

**Keywords:** gingival recession, oral hygiene, dental caries, periodontitis, smokeless tobacco

## Abstract

Introduction: The consumption of smokeless tobacco (SLT) and related products has become an epidemic worldwide, especially among young people, as they come into direct contact with the tissues of the oral cavity. Therefore, the present cross-sectional study was conducted to compare the status of dentition and periodontal health of teeth associated with the unilateral SLT pouch keratosis with the unaffected contralateral side.

Materials and methods: In this study, 96 SLT users from north Maharashtra, India, with unilateral SLT pouch keratosis were studied. Demographic data, past and present SLT use history, features of SLT pouch keratosis, modified community periodontal index, dentition status index, and loss of tooth attachment were recorded. Data were collected and subjected to statistical analysis using the unpaired t-test and chi-square test.

Results: The results of the present study showed a significant difference (p≤0.05) in gingival bleeding, pocket depth, and attachment loss in teeth associated with smokeless tobacco keratosis (STK) compared to teeth at the contralateral sides of the arch. The duration of tobacco use had a significant effect on the severity of loss of attachment at SLT pouch keratosis sides. There was a significant difference (p≤0.05) in the mean scores of the sound crown, carious crown, and coronal caries status between the SLT pouch keratosis side and the contralateral side.

Conclusion: The results of the study revealed that significant gingival bleeding, gingival recession, and attachment loss in the teeth are associated with SLT pouch keratosis compared with the teeth on the contralateral side without the lesion.

## Introduction

Periodontitis is an inflammatory illness caused by anaerobic gram-negative bacteria that damage the soft tissues and alveolar bone within the mouth [1]. However, plaque-associated microorganisms are the major etiological agents of periodontal diseases. In addition, other factors, such as malocclusion, systemic diseases, smoking, and the use of smokeless tobacco (SLT), are contributors to periodontitis [2]. Tooth decay and periodontal diseases are the most common oral diseases. Although they are accepted as unavoidable consequences of life and aging, they are largely preventable. Routine oral care combined with a healthy lifestyle and avoidance of risk factors, such as high sugar consumption and tobacco use, makes it possible to retain a functioning dentition throughout life.

Tobacco use is the most prevalent cause of death globally. The consumption of tobacco in numerous forms, such as cigarettes, pipes, cigars, and hookahs (water pipes), is predominantly linked to periodontal tissue destruction, leading to periodontitis and tooth loss [3]. Similarly, there are several types of SLT, such as loose leaves, pouches, and snuffs, that harm periodontal health [4-6].

In India, SLT products include gutkas, betel quids/pans, zarda, and khaini [6]. SLT contains several known carcinogens such as N-nitrosonornicotine (NNN), which has been proven to cause soft tissue damage. Smokeless tobacco keratosis (STK), also known as snuff dipper keratosis or tobacco pouch keratosis, develops as a result of chewing tobacco leaves or dipping snuff in contact with the oral mucosa [6,7].

According to the Global Adult Tobacco Survey (GATS) Maharashtra in 2016-2017, 31.7% of men, 16.6% of women, and 24.4% of all adults use SLT, and the prevalence of SLT use is higher in all age groups than in smoking tobacco [8]. The WHO states that the tobacco industry continues to target youth and women in developing countries; hence, the rising prevalence rates in these groups require keen attention. Several oral manifestations have been associated with SLT use at their placement sides in the oral cavity [9]. Several manifestations can occur, including oral mucosal lesions, like leukoplakia, keratosis, and oral submucous fibrosis, and gingival-periodontal soft tissue changes, such as gingival recession, gingival inflammation, changes in gingival blood flow, and loss of interproximal periodontal attachments [10].

There is insufficient literature to conclude that SLT plays a significant role in caries formation or inhibition. Unfortunately, discontinuing tobacco use does not reverse the gum problems or dental caries. Hence, it is necessary to determine whether SLTs play a role in the development of gingivitis, periodontitis, and dental caries [11]. Therefore, the present split-mouth study was undertaken to evaluate and compare the effects of SLT on periodontal health status and dentition status of teeth adjacent to the STK with the teeth on the contralateral side, where the SLT is not placed.

## Materials and methods

This in vivo, split-mouth, cross-sectional study was conducted at the Department of Public Health Dentistry. Before the start of the study, a well-defined protocol for the intended study was submitted to the Institutional Ethical Committee of ACPM Dental College, and ethical clearance was obtained (approval no. EC/INST/NEW/2022/2569/023). Written informed consent was obtained from all subjects after explaining the study.

Sample size estimation

G*Power software was used to calculate the sample size with 95% confidence and 0.05 alpha errors. The estimated sample size was 96. A total of 897 participants were screened in local camps in the district of Dhule, Maharashtra, India, to obtain a sample of 96 participants with unilateral STK. Convenience sampling was adopted for this study.

Inclusion and exclusion criteria

All young adults (males and females) aged between 18 and 35 years with dentate jaws (minimum of 20 teeth) and clinically diagnosed unilateral ST pouch keratosis with a current habit of using ST were included in the study. All the study participants should not have received oral prophylaxis in the last six months. Participants with bilateral STK, edentulous on either side of the arch, dental implants, systemic diseases, long-term medications, restricted mouth opening, or smoking tobacco history were excluded from the study.

Methodology

Data were collected from all subjects using a structured questionnaire that was self-administered by the principal investigator (PVK). The questions included information on the demographic profiles of all patients, such as age, gender, marital status, occupation, and information regarding routine oral hygiene practices (frequency, technique, and material used for brushing the teeth). Detailed information regarding past and present SLT use (brand, duration, frequency, side of placement, and time of contact with oral mucosa) was obtained. The diagnosis of ST pouch keratosis was confirmed by clinical features and stratified into degrees (grades) as given by Green et al. [12], shown in Table 1.

**Table 1:** Stratification criteria for smokeless tobacco keratosis by Greer Jr. and Poulson Citation: [12]

S.no	Stratification	Features
1	Degree 1	A superficial lesion with color similar to that of the surrounding mucosa with slight wrinkling and no obvious thickening.
2	Degree 2	A superficial whitish or reddish lesion with moderate wrinkling and no obvious thickening.
3	Degree 3	A red or white lesion with intervening furrows of normal mucosal color, obvious thickening and wrinkling.

The modified Community Periodontal Index (CPI) and Dentition Status Index (DSI) (WHO 2013) were used to assess and score variables, such as crown and root status, gingival bleeding, periodontal pocket depth, and loss of attachment [13].

For calibration, the reproducibility of the measurements performed by the same examiner was assessed by re-examining a randomly selected quadrant in 20 patients who were not included in the study. The participants in the calibration exercise underwent two examinations at 20-minute intervals during the same visit conducted by the examiner. Intra-class correlation coefficients were calculated to determine agreement between measurements. The intra-class correlation coefficients for the average pocket depth and average loss of attachment were found to be 0.89 and 0.92, respectively.

Statistical analysis

The independent variables were caries in the tooth crown and root, gingival bleeding, periodontal pocket depth, and loss of attachment. Qualitative data were presented as frequency tables, and quantitative data were analyzed as means and standard deviations. IBM SPSS Statistics for Windows, version 22 (released 2013; IBM Corp., Armonk, New York, United States) was used for descriptive and inferential statistics. Comparisons of the obtained data for independent and dependent variables were performed using parametric (unpaired T-test) and non-parametric (chi-square test) tests. Statistical significance was set at P < 0.05.

## Results

This study included 96 patients, 89.5% of whom were male and 10.5% were female, with a mean age of 29.41 ± 4.14 years. The mean duration of SLT use was 9.53 ± 5.61 years, and the mean frequency of SLT use was 6.18 ± 3.12 per day. Sixty percent of the subjects used toothbrushes to clean their teeth, whereas 36% used conventional methods, such as neem sticks and finger brushing with charcoal. Nearly 50% of the patients visited their dentists in the last year. Forty-eight percent of the population preferred chewed gutka, most commonly the Rajnigandha brand, over other SLTs. More than 60% of the subjects used to keep SLT products in their mouth for more than five minutes, as depicted in Table 2.

**Table 2:** Demographic details of the subjects SLT: smokeless tobacco

S. no.	Variables	Male	Female	Total
1	Gender n (%)	86 (89.5%)	10 (10.5%)	96 (100%)
2	Age (years)	31.23±3.12	28.23±4.02	29.41±4.14
3	Brushing habits			
	Using a brush	54 (56%)	6 (6%)	60 (62%)
	Conventional methods	32 (34%)	4 (4%)	36 (38%)
4	Brushing frequency			
	Once a day	42 (44%)	4 (4%)	46 (48%)
	Twice a day	20 (21%)	3 (3%)	23 (24%)
	Occasionally	24 (25%)	3 (3%)	27 (28%)
5	Last dental visit			
	Less than a year	36 (38%)	6 (6%)	42 (44%)
	More than a year	42 (43.5%)	2 (2%)	44 (45.5%)
	Never	8 (8.5%)	2 (2%)	10 (10.5%)
6	Type of SLT*			
	Betal quid with tobacco (paan)	25 (27%)	7 (7%)	32 (34%)
	Gutka (Rajnigandha, Vimal, Gaichaap, Goa gutka)	43 (45%)	3 (3%)	46 (48%)
	Naswar	5 (5%)	0 (0%)	5 (5%)
	Mawa/mainpuri	6 (6%)	0 (0%)	6 (6%)
	Others	7 (7%)	0 (0%)	7 (7%)
7	Duration of SLT* use (years)	11.23±2.34	7.23±3.34	9.53 ± 5.61
8	Frequency of SLT* use (per day)	7.38 ± 2.12	5.78 ± 3.32	6.18 ± 3.12
9	Retention of SLT* in mouth (minutes)			
	Less than 5 minutes	31 (32.5%)	4 (4%)	35 (36.5%)
	More than 5 minutes	55 (57.5%)	6 (6%)	61 (63.5%)

Most participants (63.5%) had STK in the right quadrant of the mandible and 34.4% in the left quadrant of mandible with a prevalence of degree 2 STK (42.7%). Therefore, STK was more prevalent in the mandible (98%) than in the maxilla (2%) due to the higher tendency of patients to keep SLT in the buccal vestibule of the mandible (Table 3).

**Table 3:** Smokeless tobacco keratosis (STK) distribution frequency in the jaw quadrants. RT: row total, CT: column total, GT: grand total

Side	Degree 1	Degree 2	Degree 3	Total	p-value (chi square)
Lower left	8 24.2% RT 32.0% CT 8.3% GT	15 45.5% RT 36.6% CT 15.6% GT	10 30.3% RT 33.3% CT 10.4% GT	33 (34.4%)	0.863
Lower right	16 26.2% RT 64.0% CT 16.7% GT	25 41.0% RT 61.0% CT 26.0% GT	20 32.8% RT 66.7% CT 20.8% GT	61 (63.5%)	
Upper left	1 50.0% RT 4.0% CT 1.0% GT	1 50.0% RT 2.4% CT 1.0% GT	0 0.0% RT 0.0% CT 0.0% GT	2 (2.1%)	
Total	25 (26.0%)	41 (42.7%)	30 (31.2%)	96	

In sound teeth, the STK side had a mean index score of 6.45 ±0.67, which was significantly lower than the mean of 7.09 ±0.93 on the contralateral side (p = 0.001). For teeth with caries, the STK side had a mean index value of 0.86 ±0.52, while the contralateral side had a mean of 0.25 ±0.88, showing a significant difference (p = 0.001). The STK side showed a statistically significant difference in the mean value of teeth filled with caries compared to the contralateral control side (p = 0.013) (Table 4).

**Table 4:** Comparison of dentition status (crown) by unpaired T-test *p value < 0.05: significant; **p value < 0.001: highly significant; NS: non-significant

Status	STK side	Contralateral side	t value	p value
Sound	6.45 ± 0.93	7.09 ± 0.67	5.541	0.001**
Caries	0.86 ± 0.88	0.25 ± 0.52	-5.892	0.001**
Filled with caries	0.00 ± 0.00	0.06 ± 0.24	-2.517	0.013*
Filled, no caries	0.00 ± 0.00	0.02 ± 0.24	-1.422	0.158^NS^
Missing due to caries	0.00 ± 0.00	0.02 ± 0.24	-1.422	0.158^NS^
Missing for any other reason	0.08 ± 0.35	0.05 ± 0.22	0.744	0.458^NS^

There was a significantly higher index score for unexposed roots on the contralateral side (6.35 ± 1.63) than on the STK side (p = 0.001). The other criteria showed non-significant differences between STK and the contralateral side, as shown in Table 5.

**Table 5:** Comparison of dentition status (root) by unpaired T-test **p value < 0.001: highly significant; NS: non-significant

Status	STK side	Contralateral side	t value	p value
Sound	0.00 ± 0.00	0.00 ± 0.00	--	--
Caries	0.02 ± 0.14	0.00 ± 0.00	1.422	0.158^NS^
Filled with caries	0.00 ± 0.00	0.00 ± 0.00	--	--
Filled, no caries	0.00 ± 0.00	0.00 ± 0.00	--	--
Unexposed root	2.71 ± 1.06	6.35 ± 1.63	-18.407	0.001**

Periodontal status assessment included gingival bleeding, pocket depth, and loss of attachment (LOA) scores. LOA scores were classified into different codes as given in the WHO index 2013. The comparison was made using unpaired T-tests, and the study demonstrated that the STK side generally exhibited more pronounced gingival bleeding but had better attachment conditions across all LOA codes than the contralateral side. However, the pocket depth did not differ significantly between the two sides (Table 6).

**Table 6:** Comparison of the periodontal status (gingival bleeding, pocket depth, and loss of attachment (LOA) scores) by unpaired T-test **p value < 0.001: highly significant; NS: non-significant

Parameters	STK side	Contralateral side	t value	p value
Gingival bleeding	5.51 ± 1.05	2.04 ± 1.63	17.561	0.001**
Pocket	0.04 ± 0.20	0.06 ± 0.32	-0.542	0.588^NS^
LOA (code 0)	2.60 ± 1.09	6.07 ± 1.75	-16.492	0.001**
LOA (code 1)	3.27 ± 1.50	1.07 ± 1.40	10.502	0.001**
LOA (code 2)	1.24 ± 1.29	0.24 ± 0.58	6.947	0.001**
LOA (code 3)	0.20 ± 0.40	0.04 ± 0.20	3.417	0.001**

The chi-square test was used to analyze the association between loss of attachment and duration of SLT use in both groups. A p-value of 0.026 indicates that there is a statistically significant difference in the loss of attachment between the two groups based on the duration of SLT use. This suggests that the duration of SLT use was significantly associated with differences in attachment loss on the STK side (Table 7).

**Table 7:** Association of loss of attachment with respect to the duration of smokeless tobacco (SLT) use by the chi-square test *p value < 0.05: significant; NS: non-significant

Groups	Duration (years)	Frequency of patients				p value
		Code 0	Code 1	Code 2	Code 3	
STK side	10 or less	0 (0%)	20 (20.8%)	18 (18.7%)	27 (28.3%)	0.026*
	More than 10	0 (0%)	2 (2%)	10 (10.4%)	19 (19.8%)	
Contralateral side	10 or less	35 (36.4%)	20 (20.8%)	8 (8.4%)	2 (2%)	0.695^NS^
	More than 10	15 (15.6%	11 (11.6%)	5 (5.2%)	0 (0%)	

## Discussion

SLT contains a variety of chemical carcinogens, including polynuclear hydrocarbon benzo[a]pyrene, nicotine, and tobacco-specific N-nitrosamines. These substances have been linked to the development of esophageal, stomach, and oral cancers and diseases of the periodontium [14-16]. SLT products available on the Indian market are likely to contain significantly higher amounts of nitrosamine. This fact was validated through a comprehensive study conducted on Indian products, which revealed that specific brands of Khaini, Zarda, and other SLT products exhibited the highest levels of tobacco-specific nitrosamines (TSNAs). The concentrations of N-nitrosonornicotine (NNN) and 4-(methylnitrosamino)-1-(3-pyridyl)-1-butanone (NNK) in these products ranged from 1.74-76.9 μg/g to 0.08-28.4 μg/g [17].

STK is a consequence of persistent and continuous frictional irritation of the oral mucosa caused by SLT [18]. The rate at which this condition forms is contingent upon factors, such as the frequency of the habit, the quantity consumed, and even the specific brand utilized. The manifestation of irritation leads to the accumulation of surplus fibrin-like substance within the submucosa and a rise in keratin synthesis, ultimately culminating in the distinctive white, wrinkled manifestation of the epithelium [2,9].

The use of SLT has been associated with various oral pathologies concentrated in a particular area of SLT application. These indications include lesions on the mucosa and teeth [19,20] and effects on the gingival and periodontal regions, such as gingival recession [15], inflammation of the gingiva [16], alterations in blood [21] to the gingiva, and loss of interproximal periodontal attachment [22].

The present study included patients with a mean age group of 29.41 ± 4.14 years. Previous literature mentions that peak SLT consumption was observed in the age group of 21-40 years. Participants aged 21-30 years were found to be at a higher risk of developing STK [23]. The use of SLT was more prevalent in males, which is in agreement with previous studies [2,6,9,10].

The investigation revealed an increased occurrence of dental caries among individuals who engaged in the act of masticating tobacco compared to those who took part in the act of smoking tobacco. Dental, missing, and filled teeth (DMFT) scores were elevated within the fraction of individuals who consumed gutka compared with other forms of tobacco consumption. The posterior region of the mandible exhibited a greater manifestation of dental caries in tobacco chewers than in tobacco smokers [18]. Although tobacco is a risk factor for increased caries, the present study showed a lower caries prevalence and root exposure on the STK side than on the contralateral side; similar results were observed in a study in which smokers were compared to SLT chewers [24]. A possible explanation for this phenomenon may be attributed to the prolonged mastication of tobacco, resulting in erosion of the occlusal surface, thereby contributing to a reduced occurrence of dental caries.

Our results showed that STK was primarily associated with the third and fourth quadrants and was rarely observed in the first or second quadrant. The second and third degrees of STK are usually found in the third and fourth quadrants. Similar findings were observed in the studies conducted by Chu et al. [10] and Bhandarkar et al. [23] The underlying cause of this phenomenon may be the inherent inclination of individuals to position SLT within the buccal vestibule of the mandible, a behavior that could potentially be acquired through observational learning from one's peers.

This study reinforces the detrimental effects of SLT use on periodontal health. The results showed that gingival bleeding, gingival recession, and attachment loss were higher in the teeth associated with STK placement than in those at the contralateral side of the arch. This implies that the vexing characteristics of snuff, whether chemical or mechanical in nature, could potentially play a role in the heightened inflammation observed at the placement sides. Conversely, the findings of Robertson et al. [15] did not reveal any correlation between the insertion of the SLT and the occurrence of gingivitis but was similar to that of Yaragani et al. [25] A side-specific study by Poore et al. [16] revealed significantly higher gingival inflammation at SLT placement sides in users than in non-users.

Because of the apparent existence of a dose-response relationship between the utilization of SLT and the level of damage inflicted upon the periodontium, the present study population was divided into different groups based on the duration of SLT use [18,19]. The loss of attachment on the STK placement side was greater in patients who underwent SLT use for less than 10 years. Similar results were reported by Katuri et al., where more attachment loss was noticed in SLT users for a shorter duration of time [26].

The selection of subjects for the current investigation was determined by the clinical diagnosis of unilateral oral STK lesions, with no supplementary data gathered from participants regarding the previous side or potential alternative selection of the SLT placement side. The small study population also provided limited data on the loss of attachment and periodontal pocket. Another limitation of the present study is the use of the CPI and DSI for evaluation, which are performed on specific teeth and do not consider all the teeth. In addition, our study used convenience sampling, which limits the generalizability of the study results. Future studies should be conducted with larger sample sizes in different parts of the country, using more parameters to evaluate the effects of SLT on dentition.

## Conclusions

Teeth associated with STK showed significantly greater gingival bleeding, gingival recession, and attachment loss than corresponding teeth on the contralateral side. The findings of the present study provide a basis for public health campaigns to screen and educate patients on the habits of using SLT. Prompt actions and raising awareness of the use of SLT can prevent the development of smokeless oral keratosis and its adverse effects on dentition.
